# Characterization, validation, and cross-species transferability of EST-SSR markers developed from *Lycori*s *aurea* and their application in genetic evaluation of *Lycoris* species

**DOI:** 10.1186/s12870-020-02727-3

**Published:** 2020-11-16

**Authors:** Yumei Jiang, Sheng Xu, Rong Wang, Jiayu Zhou, Jian Dou, Qian Yin, Ren Wang

**Affiliations:** 1 Jiangsu Key Laboratory for the Research and Utilization of Plant Resources, Institute of Botany, Jiangsu Province and Chinese Academy of Sciences, Nanjing, 210014 China; 2 The Jiangsu Provincial Platform for Conservation and Utilization of Agricultural Germplasm, Nanjing, 210014 China

**Keywords:** EST-SSR, Molecular markers, Genetic analysis, Polymorphism, *Lycoris*

## Abstract

**Background:**

The *Lycoris* genus includes many ornamentally and medicinally important species. Polyploidization and hybridization are considered modes of speciation in this genus, implying great genetic diversity. However, the lack of effective molecular markers has limited the genetic analysis of this genus.

**Results:**

In this study, mining of EST-SSR markers was performed using transcriptome sequences of *L. aurea*, and 839 primer pairs for non-redundant EST-SSRs were successfully designed. A subset of 60 pairs was randomly selected for validation, of which 44 pairs could amplify products of the expected size. Cross-species transferability of the 60 primer pairs among *Lycoris* species were assessed in *L. radiata* Hreb, *L. sprengeri* Comes ex Baker, *L. chinensis* Traub and *L. anhuiensis*, of which between 38 to 77% of the primers were able to amplify products in these *Lycoris* species. Furthermore, 20 and 10 amplification products were selected for sequencing verification in *L. aurea* and *L. radiata* respectively. All products were validated as expected SSRs. In addition, 15 SSRs, including 10 sequence-verified and 5 unverified SSRs were selected and used to evaluate the genetic diversity of seven *L. radiata* lines. Among these, there were three sterile lines, three fertile lines and one line represented by the offspring of one fertile line. Unweighted pair group method with arithmetic mean analysis (UPGMA) demonstrated that the outgroup, *L. aurea* was separated from *L. radiata* lines and that the seven *L. radiata* lines were clustered into two groups, consistent with their fertility. Interestingly, even a dendrogram with 34 individuals representing the seven *L. radiata* lines was almost consistent with fertility.

**Conclusions:**

This study supplies a pool of potential 839 non-redundant SSR markers for genetic analysis of *Lycoris* genus, that present high amplification rate, transferability and efficiency, which will facilitate genetic analysis and breeding program in *Lycoris*.

**Supplementary Information:**

The online version contains supplementary material available at 10.1186/s12870-020-02727-3.

## Background

The genus *Lycoris*, a member of the Amaryllidaceae family, contains 30 species distributed all over the world. In China, there are 15 species and 1 variety, among which, *L. radiata* and *L. aurea* are the most widespread species. Species of *Lycoris* genus are used as medical herbs that produce unique amaryllidaceae alkaloids, which exhibit a wide range of medical functions, such as anti-viral, anti-tumor and acetyl-cholinesterase-inhibitory activities [1–4]. Moreover, since flowers of this genus have a variety of colors and shapes, and some also have fragrance [5], they are very popular as ornamental plants. Hence the demand for *Lycoris* species has been increasing.

Polyploidization and hybridization are considered modes of speciation in this genus [6–9]. Karyotype investigation has been widely performed in *Lycoris*, and many karyotypes, such as 2n = 12, 13, 14, 15, 16, 17, 18, 19, 21, 22, 24, 25, 27, 30, 32,33, 44 have been found, corresponding to a status of diploid, aneuploid, triploid, and tetraploid respectively [7, 8, 10–13]. The basic chromosome number of *Lycoris* varies from x = 6 to x = 11 [5], while x = 8 and x = 11 are the main types. For example, *L. chinensis*, *L. anhuiensis* and *L. longituba* are species with x = 8, and *L. radiata*, *L. sprengeri* and *L. sanguinea* are species with x = 11 [6, 8]. Moreover, some species originated from hybridization between two species with x = 11, or between species with x = 8, and with x = 11. For example, *L. straminea* is a hybrid between *L. radiata var. pumila* and *L. chinensis* [6, 14]. Flow cytometry analysis demonstrated that genome sizes of species with x = 11 and x = 8 differ greatly and even variation was observed among lines of *L. radiata* [15]. Based on these observations, *Lycoris* species are expected to have high genetic diversity. Thus, genetic analysis of *Lycoris* is important not only for uncovering the origin of *Lycoris* species, but also for efficient conservation and development of elite germplasm.

Molecular markers have proven to be effective in the analysis of genetic diversity. Many types of DNA-based molecular markers have been employed in the analysis of genetic diversity, such as RFLPs, RAPDs and SSRs [16, 17], among which SSRs are very popular for their co-dominant inheritance, good reproducibility and cost-effectiveness [18, 19]. SSRs are developed from two types of sequence resources. One is from genomic DNA, called genomic SSRs, and the other is from expressed sequence tags (ESTs), called EST-SSRs or genic SSRs. Compared to genomic SSRs, EST-SSRs are more transferable across related species [20]. Nowadays, more and more species have been sequenced using Next Generation Sequencing techniques, thus a large number of referable ESTs are available in GenBank. Therefore, developing EST-SSR markers will be convenient for many species, especially for those whose genomic researches progress slowly. For example, EST-SSR markers have been developed from transcriptome data of *Curcuma alismatifolia* [21], *Dendrobium officinale* [22], *Salix psammophila* [23] and *Robinia pseudoacacia* [24].

In *Lycoris*, transcriptome sequencing has been performed in *L. longituba*, *L. aurea* and *L. sprengeri* [25–28], and abundant sequences containing SSRs have been obtained, which offer a convenient and cost-effective opportunity to develop EST-SSRs. Nonetheless, there have been only 27 EST-SSRs developed from *L. longituba* [25] and *L. sprengeri* [29], and 27 genomic SSRs from *L. radiata*, as well as a hybrid between *L. aurea* and *L. radiata* [30, 31]. Thus, developing more SSRs in *Lycoris* genus is demanded for genetic analysis and for facilitating research on germplasm conservation and breeding. Previously, using short reads sequencing technology (Illumina), we sequenced the transcriptome of *L. aurea* seedlings subjected to methyl jasmonate (MJ) treatment and assembled 59,643 unigenes [27]. In this study, EST-SSR mining was carried out based on these data. The major objective of this study was to supply a vast pool of non-redundant SSR markers for the genetic analysis of the *Lycoris* genus and to facilitate the development and utilization of elite germplasm in this genus.

## Results

### Development and characterization of EST-SSRs

Among the 59,643 unigenes assembled from transcriptome sequences of *L. aurea* [27], a total of 4637 SSRs were detected using the MISA program. To eliminate the redundant SSRs with respect to alternative transcripts of the same gene, sequence alignment was performed with BioEdit. Finally, primers were designed for a total of 839 SSRs including 623 tri-nucleotide repeats, 147 di-nucleotide repeats, 9 tetra-nucleotide repeats, 13 penta-nucleotide repeats, and 47 hexa-nucleotide repeats. As shown in Table 1, tri- and di-nucleotide repeats were main types of the detected SSRs, which was consistent with a previous report from an analysis using 454 pyrosequencing [26]. Our data showed that tri-nucleotide repeats accounted for 74.25%, in which (AAG)n/(CTT) n was the most common type. In addition, di-nucleotide repeats accounted for 17.52%, in which (AG)n/(CT) n repeat was the most common type, and (GC)n/(GC) n was the least common. Among the EST-SSR sequences, there were 28 ESTs containing 2 SSR loci, 4 ESTs containing 3 SSR loci and 1 EST containing 8 SSR loci. In all, a total of 839 pairs of SSR-specific primers were designed using software Primer3 (http://bioinfo.ut.ee/primer3/), representing 796 unigenes (Table S1).

**Table 1:** Frequency of di- to hexa-nucleotide repeat motifs in *L. aurea*

Types	Repeat Motifs	Numbers	Total
Dinucleotide	AC/GT	40	147
	AG/CT	77	
	AT/AT	26	
	CG/CG	4	
Trinucleotide	AAC/GTT	38	623
	AAG/CTT	116	
	AAT/ATT	20	
	ACC/GGT	36	
	ACG/CGT	32	
	ACT/AGT	35	
	AGC/GCT	41	
	AGG/CCT	47	
	ATC/GAT	37	
	ATG/CAT	26	
	CTG/CAG	45	
	CCG/CGG	39	
	TGT/ACA	7	
	TCT/AGA	33	
	TAT/ATA	5	
	GAG/CTC	41	
	GCG/CGC	12	
	GTG/CAC	13	
Tetranucleotide	AAGA	1	9
	TTTA/AAAT	3	
	ATCA	1	
	TTAT/AATA	2	
	ACAT	1	
	CATA	1	
Pentanucleotide	AAGAA	1	13
	ATTTT/AAAAT	4	
	AATTC	1	
	AAAAG/TTTTC	2	
	AAATA/TATTT	3	
	AGTGC	1	
	CCTCG	1	
Hexanucleotide	ns	ns	47

Note: *ns* not showed

### Validation of EST-SSRs

To validate the designed primers, a subset of 60 primer pairs was randomly selected and tested in *L. aurea*, which consisted of 9 di-nucleotide repeats, 46 tri-nucleotide repeats, 1 tetra-nucleotide repeats, 2 penta-nucleotide repeats and 2 hexa-nucleotide repeats. Forty-four primer pairs produced clear bands that matched the predicted sizes, accounting for 73.3% effective amplification. To confirm whether the amplification products are SSRs, 20 amplification products observed as clear and strong bands on a high-resolution agarose gel were selected, cloned and sequenced (Table S6). The results showed that the repeat motifs were consistent with the predicted sequences for all the 20 SSRs, though some SSRs varied a little in the repeat numbers. As shown in Table 2, the repeats in LaES12, LaES18, LaES25, LaES36 and LaES41 were reduced, whereas those in LaES20, LaES22, LaES31, and LaES53 were increased, while the others kept consistency with the predicted repeat number. Furthermore, the 20 sequence-verified SSRs were employed for polymorphic analysis of 11 *L. aurea* individuals (Table S2). One hundred clear bands were amplified and 85% of them were polymorphic. The results demonstrated that these EST-SSRs are useful for genetic analysis in *L. aurea*.

**Table 2:** Accuracy and authenticity of EST-SSRs confirmed by sequencing

Markers	Repeats predicted	Repeats in				
		*L. aurea*	*L. radiata*	*L. sprengeri*	*L. chinensis*	*L. anhuiensis*
LaES3	(GCA)_5_	(GCA)_5_	/	/	/	/
LaES6	(CTT)_7_	(CTT)_7_	/	/	/	/
LaES12	(GA)_8_	(GA)_6_	/	/	/	/
LaES13	(CGC)_6_	(CGC)_6_	(CGC)_4_	/	/	/
LaES18	(GAA)_5_	(GAA)_4_	(GAA)_5_	/	/	/
LaES20	(GAA)_5_	(GAA)_8_	/	/	–	/
LaES22	(AGG)_4_	(AGG)_5_	(AGG)_3_	/	/	/
LaES25	(TTCTT)_4_	(TTCTT)_3_	(TTCTT)_3_	/	–	–
LaES26	(TAT)_5_	(TAT)_5_	(TAT)_5_	/	/	/
LaES27	(TGG)_5_	(TGG)_5_	(TGG)_4_	/	/	/
LaES29	(AAGA)_5_	(AAGA)_5_	/	/	/	/
LaES31	(CTC)_5_	(CTC)_7_	/	/	–	/
LaES34	(GTT)_5_	(GTT)_5_	(GTT)_4_	/	–	/
LaES36	(CT)_8_	(CT)_7_	(CT)_7_	(CT)_6_	(CT)_7_	(CT)_8_
LaES41	(GAG)_6_	(GAG)_5_	/	/	–	–
LaES46	(AGTGC)_4_	(AGTGC)_4_	/	/	/	/
LaES49	(TA)_7_	(TA)_7_	(TA)_5_	/	–	–
LaES53	(CAT)_6_	(CAT)_7_	(CAT)_8_	(CAT)_8_	(CAT)_5_	(CAT)_5_
LaES55	(AGC)_5_	(AGC)_5_	/	/	–	–
LaES58	(ACC)_5_	(ACC)_5_	/	/	–	/

Note: ‘/’ indicated that products were not sequenced, and ‘-’ indicates no products or products with improper sizes

### Transferability of the EST-SSR markers within the *Lycoris* genus

To verify whether the primer pairs designed from the EST sequences of *L. aurea* could also effectively amplify the same SSR motifs in other *Lycoris* species. *L. radiata* and *L. sprengeri* with x = 11 as well as *L. chinensis* and *L. anhuiensis* with x = 8, were selected for analysis. As shown in Table 3, 44 of the 60 EST-SSRs amplified products of expected size in *L. aurea* and *L. radiata*, while 45, 23 and 30 EST-SSRs were amplifiable in *L. sprengeri*, *L. chinensis* and *L. anhuiensis* respectively, accounting for 38–77% amplification rate. These results demonstrated that amplification rates differed greatly in the assessed species, high in *L. radiata* and *L. sprengeri*, and low in *L. chinensis* and *L. anhuiensis*. Additionally, two markers, LaES23 and LaES24 with no amplification products in *L. aurea*, produced amplicons in *L. radiata* and/or *L. sprengeri*, indicating that an insertion may have occurred in this region of *L. aurea.*

**Table 3:** Cross-species amplification of the 60 microsatellite loci in *Lycoris* species

Markers	Amplification				
	in *L. aurea*	in *L. chinensis*	in *L. anhuiensis*	in *L. radiata*	in *L. sprengeri*
LaES1	+	+	+	+	+
LaES2	–	–	–	–	–
LaES3	+	+	+	+	+
LaES4	+	+	+	+	+
LaES5	+	+	+	+	+
LaES6	+	+	+	+	+
LaES7	+	+	+	+	+
LaES8	–	–	–	–	–
LaES9	–	–	–	–	–
LaES10	+	+	+	+	+
LaES11	–	–	–	–	–
LaES12	+	+	+	+	+
LaES13	+	+	+	+	+
LaES14	+	+	+	+	+
LaES15	+	–	+	+	+
LaES16	–	–	–	–	–
LaES17	–	–	–	–	–
LaES18	+	+	+	+	+
LaES19	+	–	–	–	–
LaES20	+	–	+	+	+
LaES21	+	–	–	+	+
LaES22	+	+	+	+	+
LaES23	–	–	–	+	+
LaES24	–	–	–	–	+
LaES25	+	–	–	+	+
LaES26	+	+	+	+	+
LaES27	+	+	+	+	+
LaES28	+	–	+	+	+
LaES29	+	+	+	+	+
LaES30	+	–	–	+	+
LaES31	+	–	+	+	+
LaES32	+	–	–	+	+
LaES33	+	–	–	+	+
LaES34	+	+	+	+	+
LaES35	–	–	–	–	–
LaES36	+	+	+	+	+
LaES37	–	–	–	–	–
LaES38	–	–	–	–	–
LaES39	+	–	–	+	+
LaES40	+	–	–	+	+
LaES41	+	–	–	+	+
LaES42	+	–	–	+	+
LaES43	+	–	–	+	+
LaES44	–	–	–	–	–
LaES45	–	–	–	–	–
LaES46	+	+	+	+	+
LaES47	+	+	+	+	+
LaES48	+	–	+	+	+
LaES49	+	–	–	+	+
LaES50	–	–	–	–	–
LaES51	+	–	+	+	+
LaES52	+	+	+	+	+
LaES53	+	+	+	+	+
LaES54	–	–	–	–	–
LaES55	+	–	–	+	+
LaES56	+	–	–	+	+
LaES57	–	–	–	–	–
LaES58	+	–	+	+	+
LaES59	+	–	+	+	+
LaES60	+	+	+	+	+
Total	44	23	30	44	45

Note: ‘+’ indicates that products with proper sizes, ‘-’ indicates no products or products with improper sizes

**Fig. 1:**
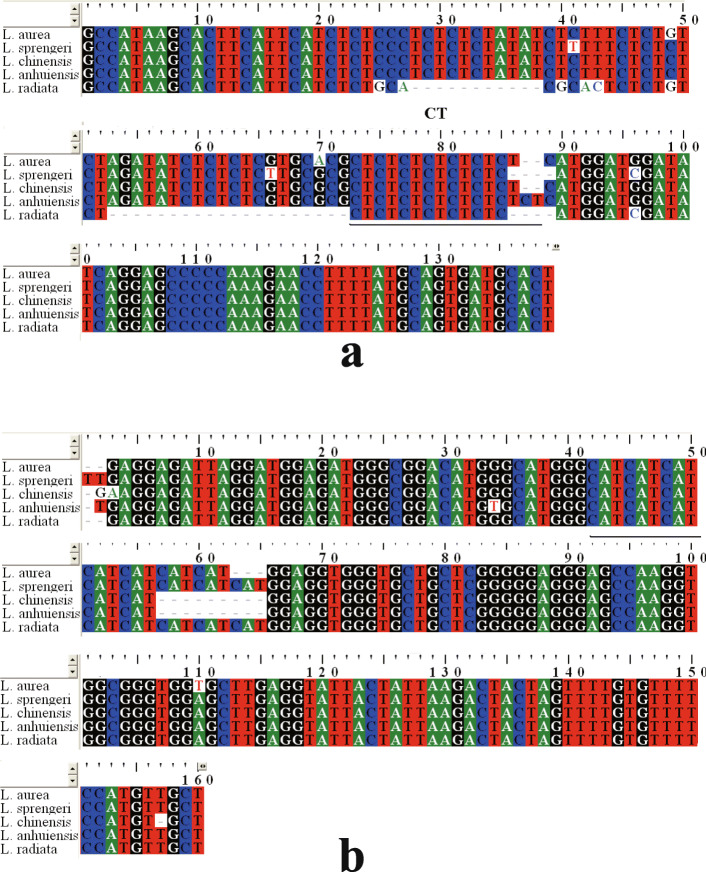
Alignment of amplicons of LaES36 and LaES53 in *L. aurea*, *L. sprengeri*, *L. chinensis*, *L. anhuiensis* and *L. radiata*. The repeat motifs are underlined. **a**, Alignment for LaES36. **b**, Alignment for LaES53

Further sequencing verification was performed to validate whether such amplification products correspond to the same SSRs in these *Lycoris* species. As shown in Table 2, ten of the 20 sequence-verified SSRs in *L. aurea* were selected and confirmed in *L. radiata*. Moreover, LaES36 and LaES53 were validated in all the five species (Table S6). In general, these repeat motifs were consistent among the *Lycoris* species, although there was a small difference in the number of repeat motifs. In the ten SSRs generated for *L. radiata*, the repeat motifs decreased in five SSRs, increased in two and were unchanged in the remaining three SSRs (Table 2). Meanwhile the sequences of LaES36 and LaES53 from all five species were aligned. As shown in Fig. 1, multiple sequence deletions were observed in the amplification of LaES36 in *L. radiata*, but the flanking sequences of the repeat motifs, designed as primer sequences, were conserved in all the five species. Conservation of the SSR flanking sequences would account for the transferability of EST-SSRs [20]. Amplification products subjected to sequence verification were all validated as SSRs, which suggested that the SSR primers with proper size products are probably authentic SSRs, further denoting high potential of the set of EST-SSRs.

### GO (gene ontology) annotation of genes harboring the set of SSRs

EST-SSRs are derived from transcribed genes and hence the annotation of the corresponding genes will be helpful for their application in trait associated marker selection [32]. GO annotations of the genes harboring EST-SSRs are listed in Table S1 according to the annotations of Wang et al. [27]. Among 839 candidate EST-SSRs, 373 have GO annotations. Of the 60 EST-SSRs, 52 have GO annotations, among which 16 EST-SSRs (LaES1, LaES19, LaES23, LaES26, LaES29, LaES31, LaES32, LaES33, LaES34, LaES35, LaES42, LaES43, LaES46, LaES47, LaES50, LaES58) are assigned to signaling or stimulus responses, 14 (LaES3, LaES16, LaES17, LaES18, LaES20, LaES22, LaES36, LaES44, LaES48, LaES51, LaES55, LaES56, LaES57, LaES59) are associated with the development of floral organs, three (LaES25, LaES49, LaES53) are involved in vegetative phase change and the remaining 19 participate in processes such as Golgi organization and cysteine biosynthesis. As the transcriptome sequences were obtained from seedlings of *L. aurea* treated with MJ, it is acceptable that many of the genes were associated with processes involved in responses to stimulus or signals.

### Genetic diversity and population structure analysis of *L. radiata* lines

To apply the newly developed SSR markers for genetic analysis, 7 *L. radiata* lines with difference in fertility, were selected for diversity analysis (Table 4). Of these, three are fertile, three are sterile, and one line consisted of the progeny of one fertile line, Pop6. Considering the GO annotations and transferability of the set of 60 EST-SSRs, 15 EST-SSR markers, containing 10 sequence-verified and 5 unverified SSR markers (LaES3, LaES20, LaES31, LaES46 and LaES58) were chosen to explore the genetic diversity of the 7 *L. radiata* lines.

**Table 4:** *L. radiata* lines used for genetic diversity analysis

Population Code	Individual Numbers	Karotype	Ploidy	Characters	Places of Collection
Pop1	3	2n = 33	triploid	sterile	Jiuhua, Jurong, Jiangsu (119° 13′ E, 32° 07′ N)
Pop2	3	2n = 22	diploid	sterile	Nanjing Botanical Garden Mem. Sun Yat-Sen (118° 83′ E, 32° 05′ N)
Pop3	4	2n = 22	diploid	sterile	Nanjing Botanical Garden Mem. Sun Yat-Sen (118° 83′ E, 32° 05′ N)
Pop4	2	2n = 22	diploid	fertile	Yixing,Wuxi, Jiangsu (119° 82′ E, 31° 34′ N)
Pop5	3	2n = 22	diploid	fertile	Pengze, Jiujiang, Jiangxi (116° 56′ E, 29° 09′ N)
Pop6	4	2n = 22	diploid	fertile	Suxian,Chenzhou,Hunan (113° 06′ E, 25° 55′ N)
Pop7	15	2n = 22	diploid	offspring	Offspring of Pop6 (118° 83′ E, 32° 05′ N)

A total of 88 bands were detected in the *L. radiata* lines, among which 80 were polymorphic. The parameters of genetic diversity and differentiation were described in Tables 5 and 6, respectively. Polymorphism information content (PIC) values of the used EST-SSR markers ranged from 0.374 to 0.850, except for LaES18 and LaES27, which demonstrated no polymorphism in the *L. radiata* lines. The mean PIC value among the verified and unverified markers were 0.636 and 0.681, respectively (Table 5). Also, the genetic differentiation (Gst) and the gene flow (Nm) parameters were similar between the verified and the unverified markers (Table 6), which suggested that both SSR groups are useful for genetic analysis in *L. radiata*.

**Table 5:** Parameters of genetic diversity among 15 EST-SSR markers for 7 *L. radiata* lines

Markers		Na	Ne	h	I	PIC
Sequence verified	LaES13	16	11.549	0.275	0.428	0.817
	LaES18	1	1	0	0	0
	LaES22	3	2.371	0.135	0.221	0.374
	LaES25	20	13.195	0.209	0.339	0.765
	LaES26	7	5.098	0.155	0.244	0.447
	LaES27	1	1	0	0	0
	LaES34	9	7.181	0.238	0.354	0.615
	LaES36	22	16.280	0.299	0.462	0.850
	LaES49	6	4.868	0.371	0.554	0.474
	LaES53	16	11.374	0.236	0.357	0.742
Mean		10.1	7.391	0.192	0.296	0.636
Sequence unverified	LaES03	12	8.181	0.254	0.415	0.708
	LaES20	18	13.527	0.300	0.455	0.828
	LaES31	17	12.168	0.373	0.390	0.801
	LaES46	7	4.812	0.153	0.262	0.603
	LaES58	13	8.451	0.130	0.217	0.466
Mean		13.4	9.428	0.242	0.348	0.681

*Na* Observed number of alleles, *Ne* Effective number of alleles, *h* Nei’s (1973) gene diversity, *I* Shannon’s Information index, *PIC* Polymorphism information content

**Table 6:** Parameters of genetic differentiation among 13 EST-SSR markers for 7 *L. radiata* lines

Markers		Ht	Hs	Gst	Nm
Verified	LaES13	0.304	0.033	0.891	0.061
	LaES22	0.168	0.036	0.786	0.136
	LaES25	0.202	0.054	0.733	0.182
	LaES26	0.133	0.046	0.654	0.264
	LaES34	0.248	0.055	0.778	0.142
	LaES36	0.300	0.061	0.797	0.128
	LaES49	0.432	0.060	0.861	0.081
	LaES53	0.220	0.059	0.732	0.183
Mean		0.200	0.040	0.779	0.147
Unverified	LaES03	0.262	0.078	0.702	0.212
	LaES20	0.325	0.052	0.840	0.095
	LaES31	0.258	0.102	0.605	0.327
	LaES46	0.200	0.058	0.710	0.204
	LaES58	0.106	0.033	0.689	0.226
Mean		0.230	0.065	0.709	0.213

*Ht* Total genetic diversity, *Hs* Within population genetic diversity, *Gst* Genetic differentiation coefficient, *Nm* Gene flow)

The average genetic differentiation parameter Gst was 0.752 among the 7 *L. radiata* lines, which means that diversity between populations accounted for 75.2% of total diversity, suggesting high genetic diversity among populations. Nevertheless, genetic communication among those *L. radiata* lines was very low, for Nm was just 0.172 (Table 6). We propose that the low genetic communication among *L radiata* lines may result from asexual reproduction, which is the main reproduction mode of *Lycoris*. Therefore, genetic diversity assessment of *Lycoris* accessions collected from different habitats is important and valuable for the development of elite germplasm.

**Fig. 2:**
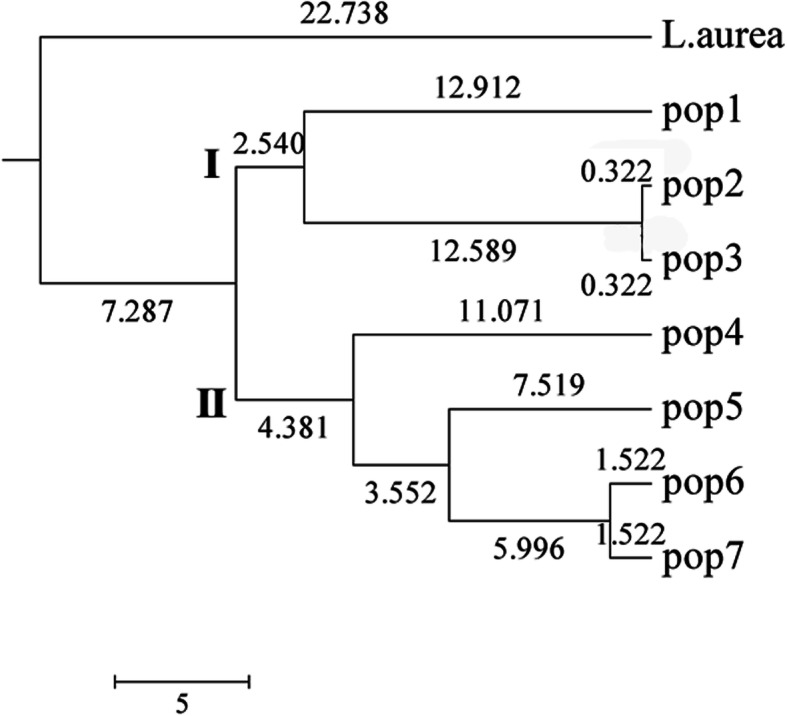
UPGMA dendrogram constructed among 34 individuals from 7 *L. radiata* lines with *L. aurea* as an outgroup based on 15 SSR markers developed in this study

In order to elucidate the genetic relationship of these 7 *L. radiata* lines, *L. aurea* was added as an outgroup to construct a dendrogram based on Nei’s genetic distance. As shown in Table 7, Nei’s genetic distances presented high variance between these lines, ranging from 0.0064 to 0.5568. The distance between *L. aurea* and Pop4 was the furthest, while that between Pop2 and Pop3 was the closest, then that between Pop6 and Pop7. Correspondingly, a dendrogram was constructed based on the genetic distance using the unweighted pair group method with arithmetic mean analysis (UPGMA). As shown in Fig. 2, the outgroup was separated from *L. radiata*, while *L. radiata* lines were clustered into two groups. Interestingly, these two groups were consistent with their fertility. The three sterile lines comprised group I, while the three fertile lines plus the offspring population formed group II. These results hinted high efficiency of the 15 EST-SSRs.

**Table 7:** Pairwise comparison of Nei’s genetic distance among populations

Pop ID		*L. aurea*	*L. radiata*						
			Pop1	Pop2	Pop3	Pop4	Pop5	Pop6	Pop7
*L. aurea*									
*L. radiata*	Pop1	0.3988							
	Pop2	0.5364	0.2666						
	Pop3	0.5316	0.2499	0.0064					
	Pop4	0.5568	0.4424	0.4327	0.4105				
	Pop5	0.5125	0.3483	0.3401	0.3219	0.2246			
	Pop6	0.3352	0.2529	0.2631	0.2397	0.2362	0.1615		
	Pop7	0.3121	0.2197	0.2284	0.2085	0.2034	0.1392	0.0304

**Fig. 3:**
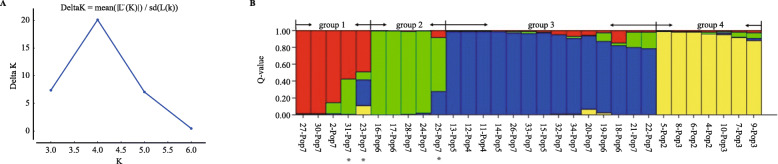
Structure analysis of *L. radiata* lines. **a** Plot of Delta K vs K values to define the most probable number of clusters in the analysis of population structure of 34 *Lycoris radiata* individuals; **b** Q values and ancestry assignment of *Lycoris radiata* individuals (Q > 0.70, K = 4). Admixed or intermediate were identified with an asterisk. The Arabic numerals represented individuals

To further evaluate the relationship of the 34 *L. radiata* individuals, population structure was analyzed with Structure 2.3.4. The ΔK method showed that the optimal K value was K = 4 (Fig. 3a), which inferred the existence of four main groups among the 34 *L. radiata* individuals. As shown in Fig. 3b, individuals from each line had the same ancestry except for lines 6 and 7, among which some individuals were scattered in more than one group, suggesting admixed ancestry of Pop6 and Pop7. From the Q value profile of populations (Table S3), it was also concluded that Pop6 and Pop7 were admixed. As shown in Fig. 3b, 6 individuals containing one from Pop6, the parent line, and five from offspring Pop7 were assigned to intermediate. It is acceptable that a parental line with an admixed ancestry would produce greater diversity in its offspring line.

## Discussion

*Lycoris* species laid a little behind other species in genetic research. So far, some molecular markers such as SCoT, ITS, have been used to determine the genetic diversity and evolutionary relationship among *Lycoris* species [33, 34]. However, these molecular markers are not sufficient for such studies as linkage group construction and marker assisted selection (MAS). Instead, SSRs are powerful DNA markers for this type of research, because of their co-dominant inheritance. Though there have been 27 genomic SSR markers and 27 genic SSR markers available in *Lycoris* species [25, 29–31], they are far from adequate for better genetic analysis of *Lycoris* genus. Since the *Lycoris* genus has a massive genome [15], a large number of DNA markers are necessary to cover the whole genome. Thus, the list of 839 potential EST-SSR markers released in this study will be helpful for genetic analysis, especially for genetic improvement of *Lycoris*.

Transcriptome sequencing technology makes it easier to develop EST-SSRs, and nowadays abundant EST-SSRs are available [35–37]. However, sequence redundancy is a major disadvantage of EST-SSRs due to multiple alternative transcripts for the same gene [21]. In addition, some SSRs occasionally exist in reverse complementary formats, but in fact they are identical. However, these common phenomenon about EST-SSRs from transcriptome sequences were often ignored in the development of EST-SSRs. Thus, the actual number of EST-SSRs obtained from transcriptome sequences should be much less than that detected. Moreover, such redundancy will reduce the representativeness of the EST-SSRs developed, and then their efficiency in application. Bazzo et al. successfully developed a total of 418 SSRs from 7492 detected EST-SSRs after considering their redundancy from transcriptome sequences of macaúba palm (*Acrocomia aculeata*) [37]. In our study, thousands of EST-SSRs were detected from transcriptome sequences of *L. aurea* seedlings treated with MJ, but many of these EST-SSRs were redundant duplicates. By eliminating duplicated EST-SSRs, we identified 839 SSRs from 4637 detected EST-SSRs as a pool of non-redundant candidate EST-SSRs, which would be promising for SSR development and correspondingly for genetic analysis.

When EST-SSRs are developed from transcribed eukaryotic genes, introns may affect the amplification products. Additionally, assembly errors may also affect amplification of the SSRs [35, 36]. Therefore, in this study, sequencing confirmation was conducted in the amplicons that generated clear bands of expected sizes, and the results confirmed that the designed primers amplified the expected loci. Supposing the same amplification rate of the subset of EST-SSRs in *L. aurea*, there would be about 615 primer pairs in the primer pool which can be developed as EST-SSR markers for *L. aurea*, suggesting great potential of the primer pool.

EST-SSRs have a high transferability among closely related species [35, 36, 38, 39]. When assaying the transferability of EST-SSRs, amplification condition affecting the effectiveness and fidelity of amplification is an important factor [19]. In this study, the annealing temperature was set at 58 °C to avoid unspecific amplification when performing PCR amplification in all the five species. In total, 23 out of 44 EST-SSRs from *L. aurea* were transferable among *L. radiata*, *L. sprengeri*, *L. chinensis* and *L. anhuiensis*, which accounted for 52.27% transferability, higher than that observed with genomic SSRs [30, 31]. Interestingly, it was found that the transferability was high in *L. radiata* and *L. sprengeri*, whereas it was lower in *L. chinensis* and *L. anhuiensis*. It is known that *L. aurea*, *L. chinensis*, *L. anhuiensis*, *L. radiata* and *L. sprengeri* are common and primitive species of *Lycoris* and also that some *Lycoris* species that originated from hybridization are mostly hybrids derived from the above species [14, 15]. Thus, EST-SSR transferability observed in this study will supply informative and practical guidance for application of this set of EST-SSRs in *Lycoris*.

The polymorphism information content (PIC) is an important parameter of a DNA marker to reflect the power of the molecular marker. In general, markers with PIC> 0.5 are defined as highly polymorphic [40]. In our case, the average PIC value of the 13 EST-SSR markers used, except LaES18 and LaES27, was 0.654, higher than many EST-SSR markers developed from transcriptome sequences, such as for *Torreya grandis* (0.357) [41], *Mucuna pruriens* (0.24) [42] and *Lagerstroemia spp* (0.589) [43]. The high PIC values observed in our work hinted a high efficiency of these set of markers in genetic analysis.

The genetic relationship of the 7 *L. radiata* lines and *L. aurea* was analyzed by using the 15 EST-SSR markers. The results showed a significant genetic distance between *L. aurea* and the *L. radiata* lines, with an average of 0.4547. The closest genetic distance, 0.0064, was found between Pop2 and Pop3, two lines from Nanjing Botanical Garden Mem. Sun Yat-Sen, which are possibly the same accessions. The Q value profile of the Structure analysis also indicated the same ancestry for Pop2 and Pop3 (Table S3). The genetic distance between an offspring line, Pop7, and its parent line, Pop6, was 0.0304, also suggesting a close relationship. In addition, the Q value profile demonstrated that both Pop6 and Pop7 had an admixed ancestry of groups 1, 2, and 3, which is reasonable for an admixed parent line can yield an admixed offspring. In general, the genetic distances and genetic structure based on the mere 15 EST-SSRs were reasonable and acceptable.

**Fig. 4:**
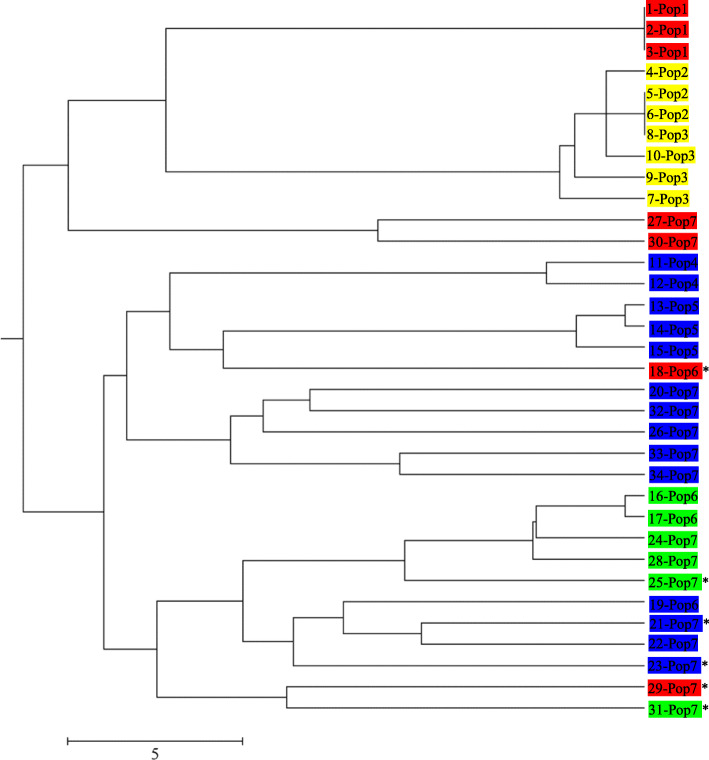
UPGMA dendrogram of the 34 *L. radiata* individuals based on 15 SSR markers. Admixed or intermediate were identified with an asterisk. Color was set consistent with structure analysis. The Arabic numerals represented individuals

Because EST-SSR markers are identified from transcribed RNA sequences and may be linked to functional genes with a possible impact on important traits, EST-SSRs may have advantages over genomic SSRs [19]. For example, González et al. focused on EST-SSRs from genes involved in some specific pathways, and three EST-SSRs were even able to discriminate different properties of the fruits [32]. In our case, the dendrogram based on mere 15 EST-SSRs showed that the outgroup, *L. aurea* was separated from *L. radiata* lines and that the 7 *L. radiata* lines were clustered into two groups consistent with their fertility. Moreover, the UPGMA dendrogram of the 34 *L. radiata* individuals based on 15 EST-SSR markers clustered all the individuals into two groups as well (Fig. 4), similar to that obtained from the analysis with the 7 *L. radiata* lines and an outgroup (Fig. 2). The first group consisted of all the individuals from three sterile lines, plus two individuals from Pop7, and another group was comprised of the remaining individuals from the fertile lines. Considering that LaES18 and LaES27 showed no polymorphism in *L. radiata* lines, the dendrogram was just based on the 13 EST-SSR markers, suggesting that those 13 EST-SSR markers are highly efficient. And the high efficiency may be associated with the functions of those transcribed genes. Although the mechanisms of fertility in *L. radiata* is unclear, many studies demonstrated that gibberellic acid (GA) and auxin signaling are involved in regulating reproductive development [44, 45]. Since crosstalk among ethylene, ABA, GA and auxin is undoubted [46], all these signals may play roles in reproductive development. Additionally, we speculated that processes of flower organ development as well as vegetative phase change, may affect seed development and finally result in a productive trait: fertility or sterility. Interestingly, among the 13 EST-SSRs, 4 are involved in hormonal signal response, 4 are associated with flower organ development and 3 participate in vegetative phase change. Furthermore, correlation analysis showed that 28 loci (LaES36–3, − 4, − 5, − 7, − 10, − 11, LaES49–2, LaES53–2, − 3, − 8, LaES58–1, LaES3–1, − 4, LaES25–4, − 5, − 7, − 8, LaES34–4, S31–4, − 5, − 6, LaES13–2, − 3, − 4, − 7, LaES20–1, − 2, − 6) from the 13 EST-SSR markers were significantly correlated with fertility (Table S5). Actually, these loci were from 10 of the above 11 EST-SSR markers, associated with flower organ development, vegetative phase change and hormonal signal response. In general, GO annotations of these 10 EST-SSRs may explain the high consistency between the clusters and fertility. Nonetheless, more work and efforts are needed to elucidate the mechanism of fertility in *L. radiata*.

## Conclusions

This study included the mining of EST-SSR markers from transcriptome data of *L. aurea*. A potential pool of 839 non-redundant EST-SSR markers was supplied for *Lycoris.* Marker characterization and validation demonstrated that the newly developed SSR markers have high amplification rate and transferability. Moreover, nearly half of the set of SSRs have GO annotations, which would be useful for trait associated marker selection in *Lycoris*. Further, 15 EST-SSRs were selected for the genetic diversity analysis of 7 *L. radiata* lines, consequently the 7 *L. radiata* lines were clustered into two groups consistent with fertility, suggesting a high efficiency of the EST-SSRs. The set of EST-SSRs would facilitate diversity analysis and breeding in *Lycoris* genus.

## Methods

### Plant materials

The plant materials include 11 *L. aurea* individuals and 34 individuals of *L. radiata*, and one individual of each *L. sprengeri*, *L. chinensis* and *L. anhuiensis*. Individuals of *L. aurea*, *L. sprengeri*, *L. chinensis* and *L. anhuiensis* were collected from Nanjing Botanical Garden Mem. Sun Yat-Sen (118°83′ E, 32°05′ N). Collection information and characteristics of *L. radiata* lines are provided in Table 4. The voucher specimens were identified by Prof. Feng Peng and deposited in the Herbarium of Institute of Botany, Jiangsu Province and Chinese Academy of Sciences. The plant materials used in this study were cultivated in the nursery of Nanjing Botanical Garden Mem. Sun Yat-Sen, Nanjing, China. Seeds of one line (Pop6) were collected from the nursery in November of 2017 and germinated in an illuminated incubator at 22 °C, 2000 lx, and a 16 h/8 h light/dark (L/D) cycle. Young leaves of the above individuals were collected for DNA extraction.

EST-SSR validation was performed in *L. aurea* (Table S2). Cross-species transferability analysis of EST-SSRs was performed in *L. radiata*, *L. sprengeri*, *L. chinensis* and *L. anhuiensis*. Thirty-four *L. radiata* individuals that belong to 7 lines were used for genetic diversity analysis (Table 4).

### SSR mining and primer design

The MISA [47] was used for microsatellites screening. Perfect di-, tri-, tetra-, penta-, and hexa-nucleotide motifs were detected by setting the parameters to a minimum of 6, 5, 4, 4, and 4 repeats, respectively. SSR sequences in different transcripts of the same gene were aligned using BioEdit to detect duplicate EST-SSRs. Primer pairs were designed using software Primer3 (http://bioinfo.ut.ee/primer3/) with the parameters set as: primer length of 18–24 bases, GC content of 40–60%, annealing temperatures of 52–60 °C, and PCR product size of 80–300 bp.

### DNA extraction

Young leaves of each individual were ground into powder with liquid nitrogen. DNA extraction was carried out according to instructions of the plant genomic DNA Mini Kit (Tiangen, Beijing, China), and DNA was detected on 1% agarose gel to evaluate DNA quality and concentration. The total DNA samples were diluted at concentration of 20 ng/μL with TE buffer and stored at − 20 °C for PCR amplification.

### EST-SSR validation

PCR reactions were carried out in a 20 μL reaction volume, containing 20 ng of genomic DNA, 0.5 μM of each primer, and 10 μL of 2x Taq Master Mix (Dye Plus) (Vazyme Biotech, Nanjing, China). The PCR reactions were performed in an Eppendorf Mastercycler ep gradient thermal cycler using the following program: 3 min at 94 °C, followed by 35 cycles of 30 s at 94 °C, 30 s at 58 °C, and 30 s at 72 °C, then a final extension at 72 °C for 5 min. PCR products were analyzed via 2% MetaPhor™ agarose (Lonza.com) gel electrophoresis, and products with strong and clear band were cloned into a T-vector and sequenced.

### Genetic diversity analysis in *L. radiata*

PCR products were separated on 8% non-denaturing polyacrylamide gels for polymorphism analysis and visualized by silver staining. PCR products were manually scored based on allele size following data scoring as “0” in the absence of the band and “1” as its presence. The binary data matrix was subjected to POPGENE1.32. Population genetic parameters of 7 *L. radiata* lines, (Na, Ne, h, I) and differentiation parameters (Ht, Hs, Gst and Nm) were evaluated by POPGENE version 1.32 [48] and PIC by PIC_CALC [49]. The UPGMA dendrogram of lines was constructed with *L. aurea* as an outgroup based on the genetic similarities, allowing a 1000 replicate bootstrap. Similarly, an UPGMA dendrogram was constructed with the 34 *L. radiata* individuals.

Statistical analyses were conducted using SPSS statistics (version 20), applying the Spearman correlation coefficient test.

### Population structure analysis

An analysis of population structure and ancestry of the 34 *L. radiata* individuals based on Bayesian statistics, without prior assignment to populations, was performed using Structure v.2.3.4 [50, 51]. In this study, SSRs were applied as dominant markers and the binary data (0, 1) were used. Since the input data in different ploidy models is not acceptable in Structure 2.3.4, Pop1, a triploid population was denoted as a diploid population. Batch runs with correlated and independent allele frequencies among inferred clusters were tested with population parameters set to admixture model (burn-in 50,000; run-length 100,000). The program Structure Harvester (http:// taylor0.biology.ucla.edu/structure Harvester/#) was used to estimate the final K value for the STRUCTURE analysis based on both the Plot of mean posterior probability (LnP(D)) values and the ad hoc Evanno’s ΔK statistics [52]. *L. radiata* individuals were allocated to a cluster if Q values were greater or equal to 0.70, or otherwise considered as intermediate or admixed.

## Supplementary Information

**Additional file 1: Table S1.** Contig sequence and primer information of 839 microsatellite markers developed for *Lycoris*.

**Additional file 2:** **Table S2.** Collection information of 11 *L. aurea* individuals.

**Additional file 3: Table S3.** Q value profile of the 7 *L. radiata* lines.

**Additional file 4: Table S4.** Q value profile of the 34 *L. radiata* individuals.

**Additional file 5:** **Table S5.** Correlation coefficients among fertility and loci.

**Additional file 6: Table S6.** Sequences of EST-SSRs validated in *Lycoris* species.

## Data Availability

All data generated or analyzed during this study are included in this published article and its supplementary information files.

## Abbreviations

SSR: Simple sequence repeats
ESTs: Expressed sequence tags
EST-SSR: Simple sequence repeats in expressed sequence tags
UPGMA: The unweighted pair group method with arithmetic means
MJ: Methyl jasmonate
MAS: Marker assisted selection
GO: Gene ontology
GA: Gibberellic acid
SCoT: Start codon targeted marker
Na: Observed number of alleles
Ne: Effective number of alleles
h: Nei’s (1973) gene diversity
I: Shannon’s Information index
Ht: Total genetic diversity
Hs: Within population genetic diversity
Gst: Genetic differentiation coefficient
PIC: Polymorphism information content
Nm: Gene flow
Pop: Population
L/D: Light/dark
