# Supplementary material for: Characterization, validation, and cross-species transferability of EST-SSR markers developed from Lycoris aurea and their application in genetic evaluation of Lycoris species
Source: BMC Plant Biol. 2020 Nov 16;20:522. doi: 10.1186/s12870-020-02727-3 (PMC7670666; doi:10.1186/s12870-020-02727-3)
Supplement: Supplementary file 2 — Additional file 2: Table S2. Collection information of 11 L. aurea individuals. [file 12870_2020_2727_MOESM2_ESM.docx]

Table S2 Collection information of 11 *L. aurea* individuals

| *L. aurea*  individuals | Collection places | Notes |
| --- | --- | --- |
| 1 | Aoshang, Huaihua, Hunan  (109°60´E, 26°59´N) | Introduced in 2013, and planted in the nursery |
| 2 | Suxian, Chenzhou, Hunan  (113°09´E, 25°60´N) | Introduced in 2013, and planted in the nursery |
| 3 | Wanhuayan, Chenzhou, Hunan  (112°95´E, 25°71´N) | Introduced in 2013, and planted in the nursery |
| 4 | Suxian, Chenzhou, Hunan  (113°09´E, 25°60´N) | Introduced in 2001, and planted in the nursery |
| 5 | Huangshi, Chenzhou, Hunan  (112°82´E,25°34´N) | Introduced in 2001, and planted in the nursery |
| 6 | Progeny of accession 5 | Seeds were harvested in 2016, and cultivated in the incubator |
| 7 |  |  |
| 8 |  |  |
| 9 |  |  |
| 10 |  |  |
| 11 |  |  |
