# Supplementary material for: Characterization, validation, and cross-species transferability of EST-SSR markers developed from Lycoris aurea and their application in genetic evaluation of Lycoris species
Source: BMC Plant Biol. 2020 Nov 16;20:522. doi: 10.1186/s12870-020-02727-3 (PMC7670666; doi:10.1186/s12870-020-02727-3)
Supplement: Supplementary file 3 — Additional file 3: Table S3. Q value profile of the 7 L. radiata lines. [file 12870_2020_2727_MOESM3_ESM.docx]

Table S3 Q value profile of the 7 *L. radiata* lines

|  | Q value | | | |
| --- | --- | --- | --- | --- |
|  | group1 | group2 | group3 | group4 |
| Pop1 | 0.8278 | 0.0098 | 0.0100 | 0.1524 |
| Pop2 | 0.0042 | 0.0042 | 0.0048 | 0.9868 |
| Pop3 | 0.0096 | 0.0356 | 0.0134 | 0.9414 |
| Pop4 | 0.0058 | 0.0076 | 0.9828 | 0.0038 |
| Pop5 | 0.0110 | 0.0086 | 0.9716 | 0.0088 |
| Pop6 | 0.1657 | 0.5244 | 0.2841 | 0.0258 |
| Pop7 | 0.2566 | 0.2616 | 0.4446 | 0.0372 |

group 1, 2, 3, and 4 are consistent with Fig. 3.
