# Supplementary material for: Characterization, validation, and cross-species transferability of EST-SSR markers developed from Lycoris aurea and their application in genetic evaluation of Lycoris species
Source: BMC Plant Biol. 2020 Nov 16;20:522. doi: 10.1186/s12870-020-02727-3 (PMC7670666; doi:10.1186/s12870-020-02727-3)
Supplement: Supplementary file 4 — Additional file 4: Table S4. Q value profile of the 34 L. radiata individuals. [file 12870_2020_2727_MOESM4_ESM.docx]

Table S4 Q value profile of the 34 *L. radiata* individuals

|  | Q value | | | |
| --- | --- | --- | --- | --- |
|  | group1 | group2 | group3 | group4 |
| Ind1 | 0.8300 | 0.0094 | 0.0098 | 0.1508 |
| Ind2 | 0.8248 | 0.0098 | 0.0104 | 0.1550 |
| Ind3 | 0.8280 | 0.0108 | 0.0098 | 0.1514 |
| Ind4 | 0.0038 | 0.0046 | 0.0052 | 0.9864 |
| Ind5 | 0.0038 | 0.0040 | 0.0048 | 0.9874 |
| Ind6 | 0.0042 | 0.0038 | 0.0052 | 0.9868 |
| Ind7 | 0.0094 | 0.0930 | 0.0050 | 0.8926 |
| Ind8 | 0.0038 | 0.0036 | 0.0046 | 0.9880 |
| Ind9 | 0.0152 | 0.033 | 0.0274 | 0.9244 |
| Ind10 | 0.0100 | 0.01240 | 0.0156 | 0.9620 |
| Ind11 | 0.0054 | 0.0070 | 0.9836 | 0.0040 |
| Ind12 | 0.0058 | 0.0078 | 0.9826 | 0.0038 |
| Ind13 | 0.0076 | 0.0062 | 0.9804 | 0.0058 |
| Ind14 | 0.0100 | 0.0068 | 0.9768 | 0.0064 |
| Ind15 | 0.0154 | 0.0128 | 0.9574 | 0.0144 |
| Ind16 | 0.0042 | 0.9860 | 0.0060 | 0.0038 |
| Ind17 | 0.0082 | 0.9708 | 0.0130 | 0.0080 |
| Ind18 | 0.6068 | 0.0248 | 0.3598 | 0.0086 |
| Ind19 | 0.0430 | 0.1158 | 0.7579 | 0.0832 |
| Ind20 | 0.0462 | 0.0166 | 0.7752 | 0.1620 |
| Ind21 | 0.0262 | 0.2748 | 0.6909 | 0.0082 |
| Ind22 | 0.0102 | 0.2581 | 0.7261 | 0.0056 |
| Ind23 | 0.3399 | 0.0664 | 0.3764 | 0.2173 |
| Ind24 | 0.0264 | 0.8606 | 0.1092 | 0.0038 |
| Ind25 | 0.0660 | 0.582 | 0.3450 | 0.0070 |
| Ind26 | 0.0108 | 0.0106 | 0.9714 | 0.0072 |
| Ind27 | 0.9722 | 0.0114 | 0.0092 | 0.0072 |
| Ind28 | 0.0178 | 0.9376 | 0.0186 | 0.0260 |
| Ind29 | 0.6793 | 0.2993 | 0.0156 | 0.0058 |
| Ind30 | 0.9706 | 0.0078 | 0.0094 | 0.0122 |
| Ind31 | 0.4647 | 0.5159 | 0.0118 | 0.0076 |
| Ind32 | 0.063 | 0.0052 | 0.8976 | 0.0342 |
| Ind33 | 0.0308 | 0.0296 | 0.9222 | 0.0174 |
| Ind34 | 0.1230 | 0.0500 | 0.7901 | 0.0368 |

Ind: individual, group 1, 2, 3, and 4 are consistent with Fig. 3.
